# Supplementary material for: Patterns of Intron Gain and Loss in Fungi
Source: PLoS Biol. 2004 Nov 30;2(12):e422. doi: 10.1371/journal.pbio.0020422 (PMC532390; doi:10.1371/journal.pbio.0020422)
Supplement: Table S1 — Also available at http://genes.mit.edu/NielsenEtAl/. (4.3 MB ZIP). [file pbio.0020422.st001.zip › NielsenEtAl/html/1369.html]

AN2940.1.NCU01324.1.MG06893.1.FG01909.1


```
 CLUSTAL W (1.82) Multiple Sequence Alignments - Introns Inserted


Sequence 1: NCU01324.1	574 aa
Sequence 2: MG06893.1	547 aa
Sequence 3: FG01909.1	560 aa
Sequence 4: AN2940.1	506 aa
Alignment Length: 604 aa
Number Identitical Residues: 214 aa
Alignment Score (without introns) 11801


MG06893.1 	MADPDMKPAPIPKS----KSTTTLTSDSEPVMTPEDSTASPSRSHPDSSSPHDNAEETCA
NCU01324.1	MADLAEPEISLPVSGLSHKSEGAPSTDGEAILTPDTPSTPPLNSSPGE---HD-------
FG01909.1 	MDD--DPKAALGQG----LKSDNISTDSEIILTPEP--ASPDNAPVDS------------
AN2940.1  	-----MDEGGVHQR----RKSDAKGGDTPELQPADMEDILDSGSDYFS------------
          	          :        .      *   : ..:        :   .            

MG06893.1 	ILPETPTLSATTPKSPR-LSRNPSFSG--SSSYQEDWEALPPLDRLTVLDLLDNFALPHQ
NCU01324.1	--PNTPTLDVTTPKSPAVLSRNSSFSGSISSTLHDEWDVFPPLDRLTVLDLLDNFALPQQ
FG01909.1 	TLLDTPVLGATTPRGSM-HSRHPSFSG--SSSNHEDWDG-PPLDRLSVLDLLENFALPQQ
AN2940.1  	----PPGSPTTRPR----LSRS-------SLSYQDDWETFPPLDQLTVFDLLDNLSLSQR
          	    .*   .* *:     **        * : :::*:  ****:*:*:***:*::*.::

MG06893.1 	LELQLGKIQKG---LSKSRQAIRARGQVARDRMVEEWRQRIP-ADEQLDRYRKRMRDSVE
NCU01324.1	LEKLQRGIHARTEKMRKSGEALKEKSAIARERMVDEWRKRVPTAEEQLERYKKRMRVNVD
FG01909.1 	LEKLQKGITAQTDKVRRSREAIKTKTQSARHRMVEEWRRRVPEADEQLDRYRKQMQHRVD
AN2940.1  	LERLQRSLNLQREKVKKQQEKLRSTSKNARDRVVGELKRRVPTADEQLEKYRRRMKTGVE
          	**     :    ..: :. : ::     **.*:* * ::*:* *:***::*:::*:  *:

MG06893.1 	KLGRRWNDTKVVTAREKVSFICGVMNIFISGYLMGGLPEYMHWWYTLQIFYFLPIRYYTY
NCU01324.1	KLGKRWNDTKVITLREKVAFIFGVMNIFISGYLIGAFPEWFHIWYTVQILYFMPIRFYRY
FG01909.1 	KLGKRWGDTKVISAREKVSFIFGVMNIFLSGYLIGAHPEQFHWWYTVQLLYFMPIRFFTY
AN2940.1  	RLSQRWNAASAVTLREKISFIAGVLNIFISGYLLGSNPEYFYIWFSAQLAYFMPIRFYRY
          	:*.:**. :..:: ***::** **:***:****:*. ** :: *:: *: **:***:: *

MG06893.1 	HRRGYHYFLADLCYFVNFLLVLSIWFFPNSKRLFTAVYCLAFGNNAVAIVMWRNSLVFHS
NCU01324.1	HKQGYHYFLADLCYFVNFLLLLSLWVFPGSKRLFTAVYCLAYGNNAIAIIMWRNSLVFHS
FG01909.1 	RQRGYHYFVADLCYFTNLLLALSIWVFPGSKRLFTASYCLAFGNNAVAIIMWRNSLVFHS
AN2940.1  	HSIGYHYFLADLCYFVNVLCMLSIWVFPQYKRLFISTFCLTFGNNAVAIAMWRNSMVFHS
          	:  *****:******.*.*  **:*.**  **** : :**::****:** *****:****

MG06893.1 	FDKVTS~LFIHIMPCATLHCMVHLIDKELQEKMFPAVYLIRHSPPGSPTAYANVISMLSW
NCU01324.1	LDKVTS~LFIHIMPCATLHCIVHLLDPAEQRKRFPSIWTIKTSPPGSPTAYANVISMLAW
FG01909.1 	FDKVTS~LFIHIMPCATLHCIVHLFSPEKQKEQFPAIWTIKNSPPGSPTAYANVVSMLAW
AN2940.1  	MDKVVS2LFIHIMPPVTLHCIVHLTPLDVMKERFPAAYEIKSSEPGSPEHFG-LLSMMLW
          	:***.* ******* .****:***      .: **: : *: * ****  :. ::**: *

MG06893.1 	STIPYAVWQLSYYFFITVRRREKIAAGRPTSFTWLRKSYSKTWIGRIVLGLPDVLQEPAF
NCU01324.1	STIPYAIWQLAYYFFITVRRRDKIAAGRPTSFTWLRKSYSKNWLGKAILNLPDSLQEPAF
FG01909.1 	STLPYIFWQLMYYFFITVRRRDKIAAGRPTSFTWLKKSYANTWLGKFVLRQPERLQEATF
AN2940.1  	ATVPYVIWQLSYHLFITVRRADKIAAGRPTSFTWLRKSYAKAWIGRLVLSLPETLQAPAF
          	:*:** .*** *::****** :*************:***:: *:*: :*  *: ** .:*

MG06893.1 	MLIQYSYAVLTMLPCPIWFYSRYASASFLMFVFAWSVYNGATYYIDVFGKRFQKELEAMR
NCU01324.1	MLIQYTYAVLTMLPCPLWFYYRWLSAVFLMGVFTWSVYNGSTYYIDVFGKRFQKELEAMK
FG01909.1 	MLIQYSYALLTMLPCPLWFLSRWASATFLMVVFTWSIYNGATYYIDVFGVRFQKELEAMK
AN2940.1  	MLIQYTYALLTMIPCPIWLWSRWASGIFITGLFILSIHNGATYYIDVFGKRFQKELEELK
          	*****:**:***:***:*:  *: *. *:  :*  *::**:******** ******* ::

MG06893.1 	AEVAKWQSHPDLGSSMGSPSMGPSHESADEAEDEALLSPEATKRRP--------------
NCU01324.1	AEVAKWQHSPDG--MVHSPTLTP-HAGAQEEENKRLGEPTKNNNNSDSNDSS--------
FG01909.1 	AEVAQWQNSPEHLPQSPTLEARPDESSANQLTQQGVAPSASTGQIPSMNEVNREKNMMGG
AN2940.1  	KDVARWQSSPEG-------AITPITPGTTAHAEEKQLNARSSRSNSD-------------
          	 :**:**  *:           *   .:    ::    .  .   ..             

MG06893.1 	----------RDSVNSISLLS-------GDAEILATG--VDSSAKDVARERK--------
NCU01324.1	-------GSSTSSLDNIPLLTEEKSELPASATATATGAEVDGGAKDVARARRPGAELFGA
FG01909.1 	TSLSKDDANRPISVDNIPLLN-------DMQSSSSTG--VDGRPVNNTRGRRVD------
AN2940.1  	----------NASIEKIPLLD---------SNGVSTA--IEGGTK---------------
          	            *::.*.**              :*.  ::. .                

MG06893.1 	-----
NCU01324.1	GAAPS
FG01909.1 	-----
AN2940.1  	-----
          	
```
